# A Review of the Precision Glass Molding of Chalcogenide Glass (ChG) for Infrared Optics

**DOI:** 10.3390/mi9070337

**Published:** 2018-07-02

**Authors:** Tianfeng Zhou, Zhanchen Zhu, Xiaohua Liu, Zhiqiang Liang, Xibin Wang

**Affiliations:** 1 Key Laboratory of Fundamental Science for Advanced Machining, Beijing Institute of Technology, Beijing 100081, China; liangzhiqiang@bit.edu.cn (Z.L.); cutting0@bit.edu.cn (X.W.); 2 School of Mechanical Engineering, Beijing Institute of Technology, Beijing 100081, China; zhuzhanchen@163.com (Z.Z.); liuxh89@126.com (X.L.)

**Keywords:** chalcogenide glass, infrared optics, precision glass molding, aspherical lens, freeform optics

## Abstract

Chalcogenide glass (ChG) is increasingly demanded in infrared optical systems owing to its excellent infrared optical properties. ChG infrared optics including ChG aspherical and freeform optics are mainly fabricated using the single point diamond turning (SPDT) technique, which is characterized by high cost and low efficiency. This paper presents an overview of the ChG infrared optics fabrication technique through precision glass molding (PGM). It introduces the thermo-mechanical properties of ChG and models the elastic-viscoplasticity constitutive of ChG. The forming accuracy and surface defects of the formed ChG are discussed, and the countermeasures to improve the optics quality are also reviewed. Moreover, the latest advancements in ChG precision molding are detailed, including the aspherical lens molding process, the ChG freeform optics molding process, and some new improvements in PGM.

## 1. Introduction

### 1.1. Introduction of ChG Infrared Optics

#### 1.1.1. Characteristics of ChG

Infrared optical systems, including thermal imaging devices and night vision cameras, are gaining more attention in the optical fields [1,2]. Crystalline infrared materials such as single-crystal germanium (Ge) and zinc selenide (ZnSe) have been used and primarily fabricated by single point diamond turning (SPDT) and chemical vapour deposition (CVD) in past decades, though they are rare and expensive [3,4,5]. Chalcogenide glass (ChG), which is a kind of artificial material mainly made of chalcogens (S, Se, and Te) and some other elements, shows much wider transmission wave band from near to far infrared wavelength compared with the oxide glasses and has more excellent properties of athermalization and achromatism compared with the crystalline infrared materials [6,7]. ChG infrared optics could be formed in mass production using the precision glass molding (PGM) process with low-cost compared to the silicon (Si) and germanium (Ge) optics machined by the diamond turning or grinding. Hence, ChG is now deemed as an alternative for crystalline infrared materials for various infrared optics [8].

ChG can be divided into sulfide glass, selenide glass, and telluride glass according to the elemental composition. The As–S and Ge–S glass are the first sulfide glasses to be studied [9,10]. As–S glass has the advantages of good infrared transmittance, high refractive index, low sound speed, and high quality factor. However, its shortcomings, such as high intrinsic loss, poor chemical stability, and so on, hinder its application in the optical industry. In order to improve the comprehensive properties of As–S glass, the preparation and properties of Ge–As–S glass have been studied. The results show that the density, hardness, softening temperature, and chemical stability of glass increase and the expansion coefficient decreases with the increase of element Ge content. The transmittance range of Ge–As–S glass is 0.6–11 μm [11].

The preparation of selenide glass is easier than sulfide glass due to the higher rate of chemical reaction between Se and other elements, as well as lower pressure when Se melts [12,13,14,15]. The Ge–As–Se glass and Ge–Sb–Se glass are the most suitable selenide glasses for infrared optical system [2,16], because Ge–As–Se glass has intrinsic optical stability and a wide temperature range of glass forming process. It has a great transmittance of far infrared wave, the range of which is 0.8–15 μm. Besides, the value of the third-order optical nonlinearity of Ge–As–Se glass is the largest among all ChGs.

Telluride glass has a wider infrared transmittance than sulfide glass and selenide glass. The infrared transmission spectrum of telluride glass can be extended above 20 μm [17,18,19]. However, the glass forming ability of Te is weak, because it has stronger metallicity compared with S and Se. The telluride glass has low glass transition point, poor thermal stability, and mechanical strength, which limits its applications in far infrared optics [20,21].

#### 1.1.2. Application of ChG Infrared System

ChG has been widely used in various infrared optical systems due to its excellent optical properties [22,23,24,25]. ChG infrared night vision system uses infrared light to enable human eyes to observe scenes at night by converting the invisible infrared image of the scene into a visually sensible image. ChG has a lower temperature dependency of the refractive index compared with single-crystal germanium (Ge) and zinc selenide (ZnSe). The night vision system designed with ChG can realize the temperature self-adaptation control and make the system image useable in a large temperature range [26].

**Figure 1:**
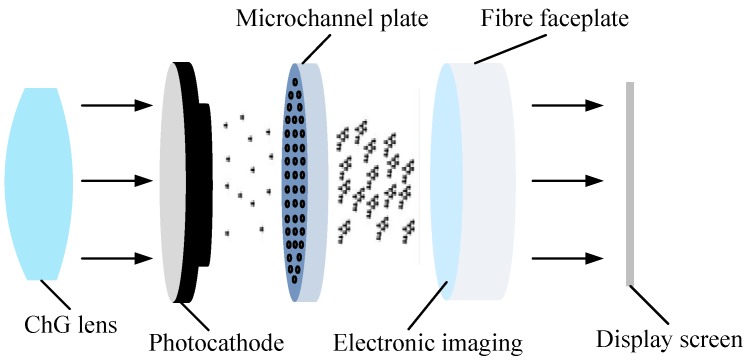
Schematic diagram of night vision system.

The ChG infrared night vision system consists of an infrared optical imaging device and a photoelectric conversion device, as shown in Figure 1. The ChG lens is responsible for transmitting infrared radiation of the targets. The photoelectric conversion device converts the infrared radiation into the visible image for human eyes, and then the image is projected on the display screen after denoising, reshaping, and amplifying [27]. The image quality is related to the shape accuracy, surface roughness, and infrared transmittance of ChG lens.

**Figure 2:**
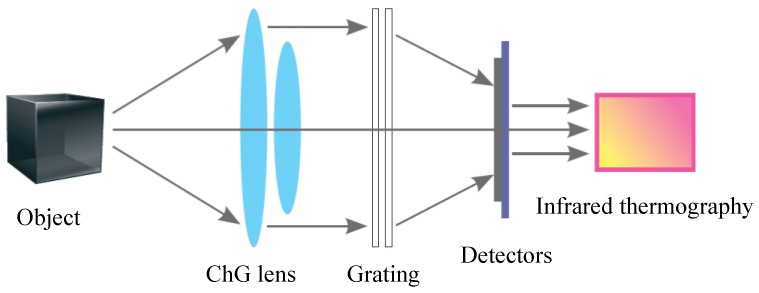
Schematic diagram of thermal imaging system [30].

ChG is also extensively applied in infrared thermal imaging technology [28,29]. Figure 2 shows the infrared thermal imaging system. The infrared detector and ChG lens are used to accept the infrared radiation energy distribution of measured target, which reflects in the photosensitive element of infrared detector to obtain infrared thermography. The infrared thermal imaging system can transform the invisible infrared radiation of the object into the visible thermal image, and different colors in the thermal image represent the different temperatures corresponding to the heat distribution of the measured object.

#### 1.1.3. Classification of ChG Infrared Optics

**Figure 3:**
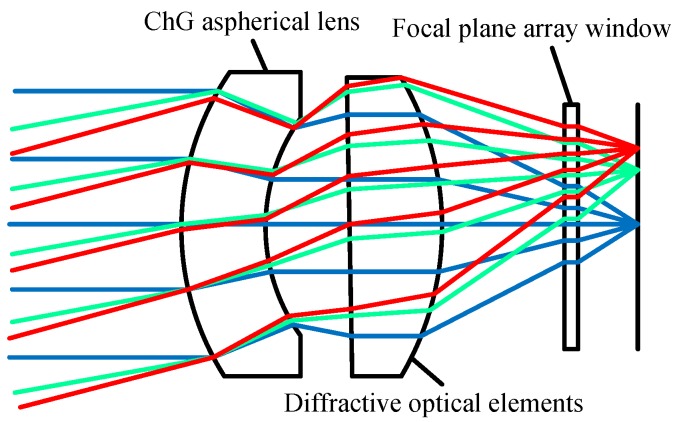
The optical layout of ChG aspherical lens.

ChG infrared optics/lenses are usually designed with complex geometric shapes to obtain superior optical performance, including the ChG aspherical lens and ChG freeform optics. ChG aspherical lens can significantly improve the image quality and infrared optical properties. As shown in Figure 3, it can change the light path to reduce the number of infrared optical elements, thereby simplifying the structure of infrared system and reducing the volume of the infrared system [31,32].

**Figure 4:**
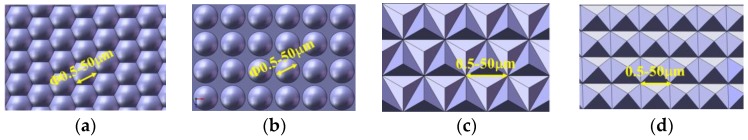
Element shapes of the ChG freeform optics: (**a**) adjacent microlens arrays, (**b**) distributed microlens arrays, (**c**) triangular pyramid arrays, and (**d**) rectangular pyramid arrays [34].

ChG freeform optics are generally defined as an infrared element with microlens array or microstructure array on the surface (shown in Figure 4). The surface microstructures of the infrared optical elements can improve the infrared thermal imaging quality and eliminate light aberration, and the microstructure array is widely used to simplify the infrared system and meet specific requirements. For example, the detection capability of infrared detectors can be improved by coupling ChG microlens array with infrared detector. At the same time, ChG microlens array has a cold shield effect. The incident angle of infrared radiation is limited to ensure that each photosensitive element of the infrared detector has the same incident angle and background radiation, and then the noise can be reduced [33].

### 1.2. Methods of ChG Optics Manufacturing

In order to meet the application requirements of ChG optics, various fabrication techniques have been developed. The conventional methods for manufacturing ChG aspherical lens are single point diamond turning and polishing. They are relatively mature and can obtain ultraprecision surface accuracy. However, they are time-consuming processes and are very ill-suited to mass production with high accuracy [35].

There are also some processes to produce ChG freeform optics. Based on energy assisted machining, the focused ion beam machining and laser processing can fabricate surface microstructures directly on optical components. However, these two methods are complex in processing, high in cost, and poor in microstructures uniformity [36,37,38]. The ChG freeform optics can also be processed by photolithography, but the shape and size of the microstructures are limited [39]. Using traditional material removal machining techniques such as single point diamond turning or ultraprecision grinding, complex surface microstructures can be fabricated. The surface microstructures have ultraprecision shape accuracy. However, these methods still have many problems such as high cost, poor efficiency, and so on. They are not capable enough to meet the needs of the market [40]. PGM can solve the problems of the processing technology mentioned above, and it is one of the most promising technologies with which to manufacture ChG optics.

### 1.3. Precision Glass Molding of ChG

PGM was earliest used to process optical aspherical lens, replacing traditional Polymethyl Methacrylate (PMMA) resin optics to achieve better optical characteristics and stability [41]. It has high processing efficiency and can achieve mass production to reduce processing cost with good formation accuracy of optical components [42,43]. Compared with single-crystal germanium, ChG is an amorphous material without a fixed melting point. The viscosity of ChG gradually decreases during heating, and it is suitable for ChG optics to be fabricated by PGM [44]. From the viewpoint of fabrication cost and process time, the PGM is undoubtedly a better approach for producing precision ChG aspherical lens and ChG freeform optics. PGM of ChG were first reported by Zhang et al. at the French company Umicore IR Glass S.A. in 2003 [44]. They produced ChG lenses by using Ge_22_As_20_Se_58_ and Ge_20_Sb_15_Se_65_. The maximum shape errors of their molded lenses were approximately 0.3 μm and 2 μm, respectively. In the same year, Zhang et al., at the University of Rennes I [3], reported a similar result that the shape error of the molded Ge_22_As_20_Se_58_ lens was approximately 0.4 μm. In 2006, Curatu et al. [29] designed and fabricated an athermalization lens over the entire operating temperature range (−40 °C~+80 °C). Cha et al. [45] studied the effect of temperature on the molding process of Ge_10_As_40_Se_50_ in 2010. They suggested that the ChG should be molded at a temperature higher than softening temperature to prevent breakage. In 2011, Liu et al. [46] conducted PGM numerical simulations for Ge_33_As_12_Se_55_ to investigate the variations of its thermos-mechanical properties. Ju et al. [35] characterized the moldability of ChG and selected the preferential Ge/Sb ratio in the Ge–Sb–Se based ChG system to fabricate lenses in 2014. Zhou et al. [47] evaluated the stress relaxation behavior of As_2_S_3_ and calculated its refractive index change in 2017.

**Figure 5:**
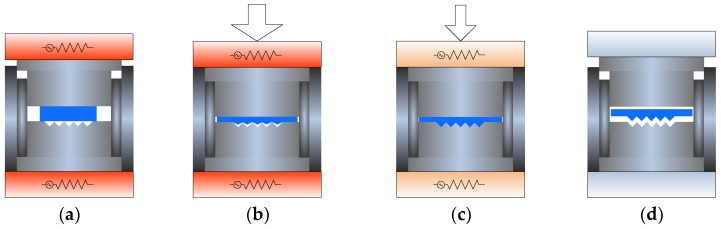
Four stages of a PGM cycle: (**a**) heating, (**b**) pressing, (**c**) annealing, and (**d**) cooling.

ChG molding process can be divided into four stages: heating, pressing, annealing, and cooling, as shown in Figure 5. In a PGM cycle, the molds and ChG are heated simultaneously. The ChG is heated above softening point and fully softened. Then, the upper mold is moved down to compress the ChG preforms, and the stress in ChG is relaxed by holding the pressing load without further deformation of ChG for a short time. After that, the ChG is annealed at a slow cooling rate. Finally, the formed ChG optics are cooled to ambient temperature and released from the molds. In this way, the geometric shapes of the molds are replicated to the ChG surfaces.

Though PGM for optical glasses is relatively established, it is still in early stages for ChGs. Directly using the constitutive model of oxide glass to describe the thermo-mechanical behavior of ChG could be problematic, because the molding temperature for oxide glass is between the yielding point and the softening point, while the molding temperature is above the softening point for ChG [45,46]. In contrast to oxide glass, most ChG has a greater saturated vapor pressure, which enables trace gases to release during the molding process. If these gases cannot escape, the lens shape and surface quality will be severely impaired [48].

## 2. Modeling and Simulation of ChG Molding

### 2.1. Modeling of Elastic-Viscoplasticity Constitutive of ChG

#### 2.1.1. Thermo-Mechanical Behavior Test of ChG

**Figure 6:**
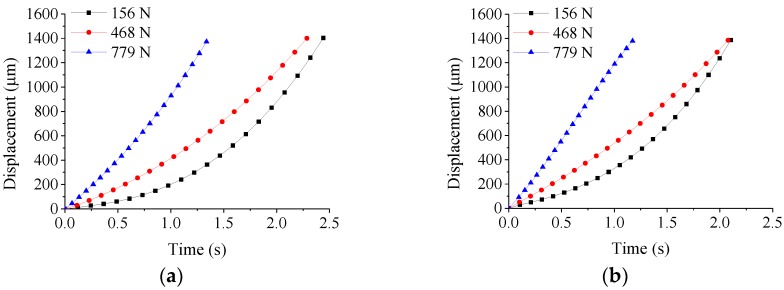
The upper mold displacement *w* during compression test under different pressing forces at the following molding temperatures: (**a**) 382 °C and (**b**) 392 °C.

Finite element method can solve issues when some variables are difficult to measure in experiments. However, a reliable simulation model requires an accurate constitutive of materials. The thermo-mechanical behavior is the basis for modelling constitutive of the ChG. The thermo-mechanical behavior test of the ChG is mainly applied in the pressing stage. The true strain and stress of the ChG are calculated by measuring the displacement curve of the upper mold. Figure 6 [49] shows the displacement *w* of the upper mold under different pressing forces in which the *w* is defined as *w*(t) = *y*(t) − *y*(0), and *y*(t) is the position of the upper mold.

**Figure 7:**
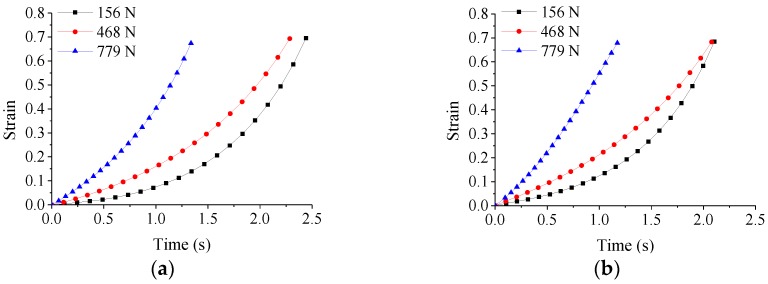
The variations of strain with time under different pressing forces at the following molding temperatures: (**a**) 382 °C and (**b**) 392 °C.

The true strain *ε_t_*, also named as logarithmic strain or Hencky strain [50], considering as an incremental strain, can be derived by Equation (1):

(1) $$\varepsilon \left(t\right)=\int_{z_{0}}^{z_{t}}\frac{dz}{z}=\ln(1-\frac{w(t)}{z_{0}})$$

in which *z*_0_*, z_t_* are the initial and instantaneous heights of the cylindrical specimen. Both stress and strain should be negative in compression but are taken as absolute values in the following figures for convenience. The changes of strain with time during deformation under different pressing forces are shown in Figure 7 [49].

**Figure 8:**
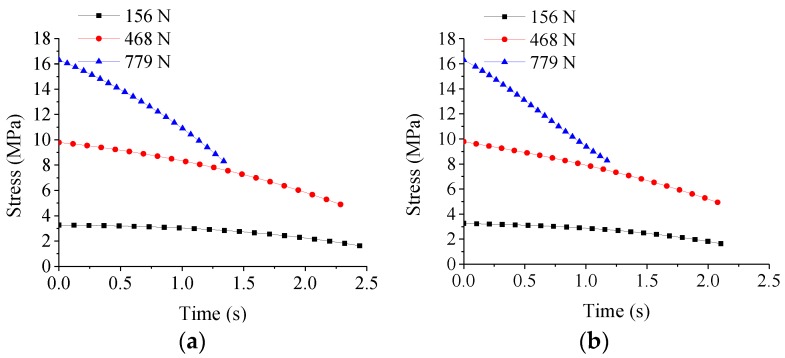
The variations of stress with time under different pressing forces at the following molding temperatures: (**a**) 382 °C and (**b**) 392 °C.

The true stress *σ*(*t*) can be derived by Equation (2):

(2) $$\sigma \left(t\right)=\frac{F}{\pi \rho_{0}^{2}}e^{\varepsilon \left(t\right)}$$

in which *F* is the molding force, and *ρ*_0_ is the radius of ChG preform. The changes of stress with time under different pressing forces are shown in Figure 8 [49]. It is noted that the stress decreases more rapidly under higher pressing force.

#### 2.1.2. Elastic-Viscoplasticity Modeling

At the molding temperature, ChG exhibits elastic-viscoplastic behavior, which is a combination of viscoelastic and viscoplastic mechanisms. As ChG is an isotropic material, the elastic-viscoplastic behavior of which can be simplified as a combination of spring, dashpot, and slider, representing elasticity, viscosity, and plasticity, respectively. The constitutive relations of these three elements are, respectively, listed as below:

(3) $$\sigma_{e}=E\varepsilon_{e}$$

(4) $$\sigma_{v}=\eta {\dot{\varepsilon }}_{v}$$

(5) $$\sigma_{p}=\sigma_{f}\mathrm{sign}\left({\dot{\varepsilon }}_{p}\right)$$

in which *σ_e_*, *σ_v_*, and *σ_p_* are the elastic stress, viscous stress, and plastic stess, respectively; *σ_f_* is the yield stress of the friction element; *ε_e_*, *ε_v_*, and *ε_p_* are corresponding strains; *E* and *η* are elastic modulus and viscosity; and $\mathrm{sign}\left({\dot{\varepsilon }}_{p}\right)$ is sign function, being equal to 1, 0, and −1 when the plastic strain rate ${\dot{\varepsilon }}_{p}$ is positive, zero, and negative.

**Figure 9:**
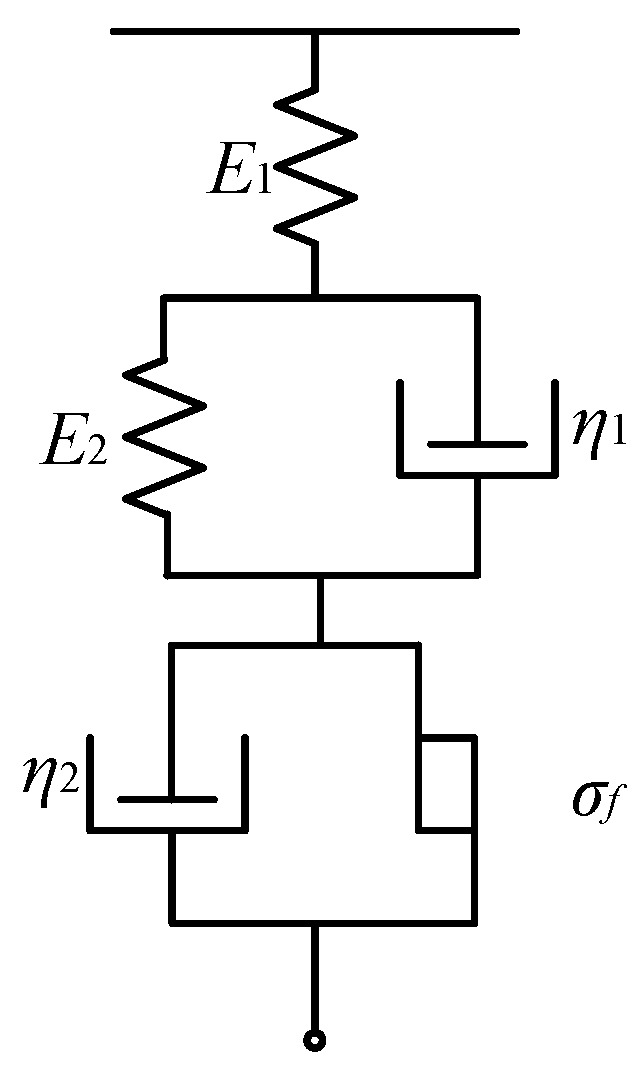
The Schofield–Scott Blair constitutive model.

In order to describe all deformation mechanisms of the ChG, Schofield–Scott Blair model is used [51], as shown in Figure 9. The instantaneous elastic and plastic deformation, delayed elastic and plastic deformation, and viscous flow coexist in this model. The constitutive equation based on the Schofield-Scott Blair model is

(6) $$\left\{ \begin{array}{l} \frac{\eta_{1}}{E_{2}}\dot{\varepsilon }+\varepsilon =\frac{\eta_{1}}{E_{1}E_{2}}\dot{\sigma }+\frac{E_{1}+E_{2}}{E_{1}E_{2}}\sigma ,\sigma <\sigma_{f}\\ \frac{\eta_{1}}{E_{2}}\ddot{\varepsilon }+\dot{\varepsilon }=\frac{\eta_{1}}{E_{1}E_{2}}\ddot{\sigma }+\frac{1}{E_{2}}\left(1+\frac{E_{2}}{E_{1}}+\frac{\eta_{1}}{\eta_{2}}\right)\dot{\sigma }+\frac{1}{\eta_{2}}\sigma -\frac{\sigma_{f}}{\eta_{2}},\sigma \geq \sigma_{f} \end{array}\right.$$

in which *E*_1_, *E*_2_ are elastic moduli and *η*_1_, *η*_2_ represent the viscosities of the elastic-viscoplastic material. As the ChG preform is compressed at a uniform temperature, the pressing stage can be treated as isothermal process without heat transfer [52].

Considering the small strain scenario, the first order Taylor expansion of Equation (2) leads to

(7) ε(t)=Cσ(t)−1

in which $C=\pi \rho_{0}^{2}/F$ is a constant. Substituting Equation (7) into Equation (6), and considering the cases of *σ* ≥ *σ_f,_* results in [49]:

(8) $$\begin{array}{l} \sigma (t)=\sigma_{f}+C\eta_{2}+\{[(E_{1}\eta_{1}+E_{1}\eta_{2}+E_{2}\eta_{2}+C_{1})(\sigma_{0}-\sigma_{f})-CC_{1}\eta_{2}-CE_{1}\eta_{1}\eta_{2}+2\eta_{1}\eta_{2}{\dot{\sigma }}_{0}\\ -C_{3}-C_{2}]e^{-\frac{\left(E_{1}\eta_{1}+E_{1}\eta_{2}+E_{2}\eta_{2}-C_{1}\right)t}{2\eta_{1}\eta_{2}}}\}/2C_{1}-\{[(E_{1}\eta_{1}+E_{1}\eta_{2}+E_{2}\eta_{2}-C_{1})(\sigma_{0}-\sigma_{f})+CC_{1}\eta_{2}-\\ CE_{1}\eta_{1}\eta_{2}+2\eta_{1}\eta_{2}{\dot{\sigma }}_{0}-C_{3}-C_{2}]e^{-\frac{\left(E_{1}\eta_{1}+E_{1}\eta_{2}+E_{2}\eta_{2}+C_{1}\right)t}{2\eta_{1}\eta_{2}}}\}/2C_{1}\begin{array}{cc} , & \end{array}\sigma \geq \sigma_{f} \end{array}$$

in which $C_{1}=\sqrt{E_{1}{}^{2}\eta_{1}{}^{2}+2E_{1}{}^{2}\eta_{1}\eta_{2}+E_{1}{}^{2}\eta_{2}{}^{2}-2E_{1}E_{2}\eta_{1}\eta_{2}+2E_{1}E_{2}\eta_{2}{}^{2}+E_{2}{}^{2}\eta_{2}{}^{2}}$, $C_{2}=CE_{2}\eta_{2}{}^{2}$, $C_{3}=CE_{1}\eta_{2}{}^{2}$, and $\sigma_{0}$ is the initial stress when the upper mold just contacts the ChG surface and ${\dot{\sigma }}_{0}$ is the stress rate at *t* = 0. The values of $\sigma_{0}$ and ${\dot{\sigma }}_{0}$ can be obtained from Figure 8, and they can be utilized in Equation (8) to determine the constitutive model parameters from compression tests mentioned above.

Considering for the large strain, the constitutive equation is

(9) $$\frac{\eta_{1}}{E_{1}E_{2}}\ddot{\sigma }+\frac{1}{E_{2}}\left(1+\frac{E_{2}}{E_{1}}+\frac{\eta_{1}}{\eta_{2}}\right)\dot{\sigma }+\frac{1}{\eta_{2}}\sigma -\frac{1}{\sigma }+\frac{\eta_{1}}{E_{2}\sigma^{2}}-\frac{\sigma_{f}}{\eta_{2}}=0,\begin{array}{cc} & \sigma \geq \sigma_{f} \end{array}$$

### 2.2. Simulation of Molding Process of ChG Infrared Optics

#### 2.2.1. Simulation of Molding for Aspherical Lens

**Figure 10:**
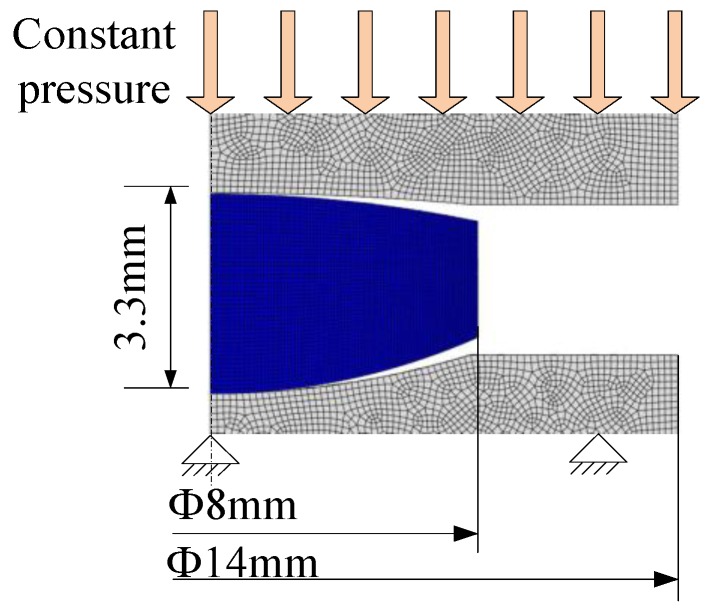
A half simulation model of ChG aspherical lens molding [49].

**Figure 11:**
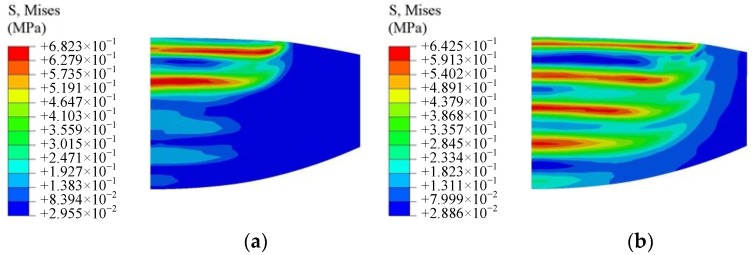
Stress distribution of ChG aspherical lens molding: (**a**) early stage and (**b**) later stage.

Based on the thermo-mechanical behavior test of ChG and Schofield-Scott Blair model, the two-dimensional (2D) axisymmetric simulation model of aspherical lens is created. In the model, three blocks are assembled from top to bottom representing upper mold, ChG preform, and lower mold, respectively, as shown in Figure 10 [49]. The lower mold is fixed, and a constant pressing force is applied on the upper mold to press the ChG with initial field temperature for the entire model of 25 °C. The distribution of equivalent stress in course of PGM is shown in Figure 11 [49].

The contact area gradually increases from point contact, and the distribution of stress is initially localized near the contact region, as shown in Figure 11a. The stresses are significantly large at this early stage of aspherical lens molding. With pressing going on, the stress decreases when the contact area becomes larger, as shown in Figure 11b. Therefore, the material flow and stress changes in PGM are obtained to predict the forming accuracy of aspherical lens.

#### 2.2.2. Simulation of Molding for Freeform Optics

**Figure 12:**
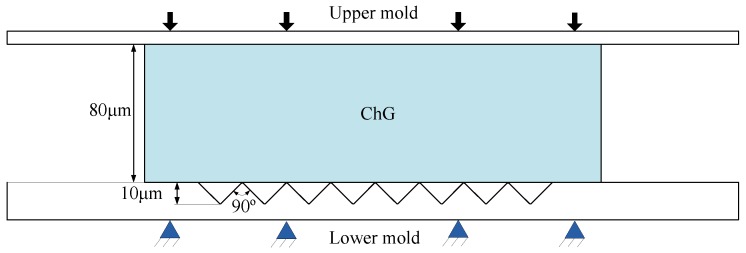
A two-dimensional simulation model of microstructures molding [30].

The molding conditions for freeform optics at different process parameters are optimized. The two-dimensional simulation model is shown in Figure 12. The flat upper mold moves downward to press the softened ChG. The lower mold with microstructures is fixed. In order to avoid the stress convergence in the contact region and save remeshing time at the sharp corners during the molding simulation, the sharp corners of the microstructures are rounded with a small radius of 0.5 μm. As the ChG preform and the molds are pressed at a uniform temperature, the pressing stage can be treated as isothermal process without heat transfer.

**Figure 13:**
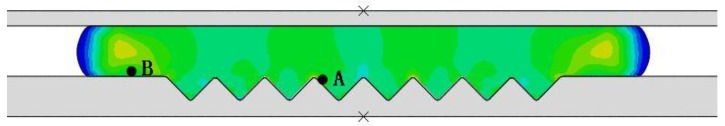
Two representative points of microstructures simulation [30].

Two representative points are selected in the simulation model of the ChG, as shown in Figure 13. The point A is at the interface between the ChG and the top of the mold microstructures. Point B is at the interface between the ChG and the mold surface without microstructures.

**Figure 14:**
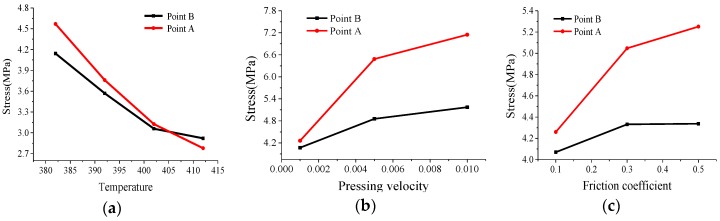
Effect of process parameters on stresses of ChG: (**a**) temperature, (**b**) pressing velocity, and (**c**) friction coefficient [30].

Figure 14 illustrates the equivalent stress changes of point A and point B at different temperatures, pressing velocities, and friction coefficients. When the temperature of the ChG rises continuously, the internal stress of ChG decreases gradually. The stresses of point A and point B increase gradually with the increase of pressing velocity and friction coefficient.

## 3. Molding Process of ChG for Aspherical Lens

### 3.1. ChG Molding Condition Optimization

**Figure 15:**
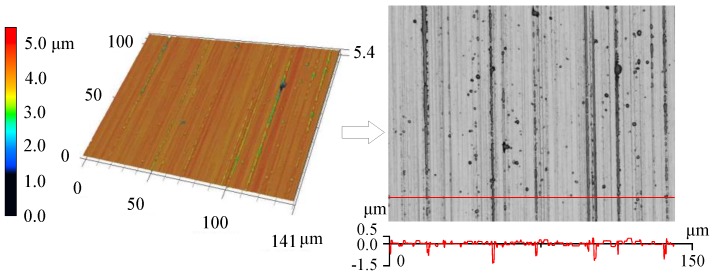
The surface morphology and contour of a formed ChG pillar.

The ChG molding parameters can be partly determined by the FEM simulation results, and the cylindrical molding tests can be conducted to explore the other molding conditions. Most ChGs have a greater saturated vapor pressure, which enables the release of trace gases during the molding process. When these gases cannot escape, microdimples will be formed on the ChG pillars. The maximum peak-to-valley height difference of microdimples is 1.562 μm, and the shape and surface quality of lens are severely impaired (in Figure 15). Therefore, the gas release and gas escape must be controlled [53].

For gas generation, the solubility of gas has been studied, and it can be expressed using A. Sieverts’ square root law [54]:

(10) $$S=k\sqrt{P}e^{-\Delta H/\left(RT\right)}$$

in which *S* is the dissolved concentration, *k* is a constant, *P* is the gas partial pressure, Δ*H* is the dissolution heat, *R* is the gas constant, and *T* is the temperature.

The movements of the molecules become more intense as the temperature increases, leading to more reactions, smaller gas solubility, and more gas generation. Pressure is another contributor that affects gas solubility. The gas density grows with the increasing pressure, so the free molecular motion decreases, and the gas increasingly dissolves, as expressed by Equation (10). According to Dalton’s law of partial pressure, the total pressure is equal to all partial pressures of the mixed gas [55]. Meanwhile, based on Newton’s third law [56], the total pressure increases with the increase of the pressing force.

**Figure 16:**
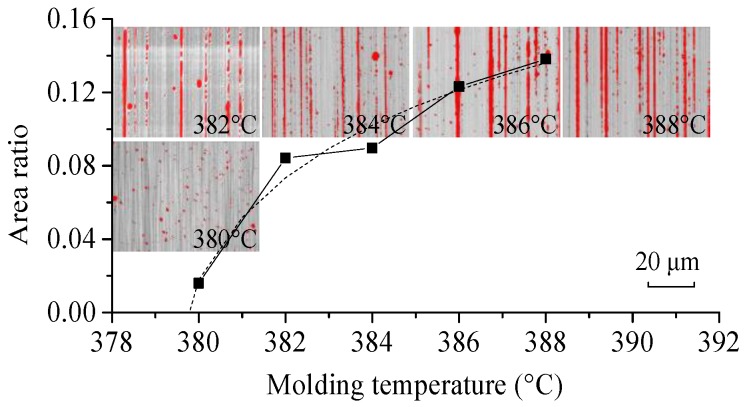
Area ratios of the microdimples of the formed ChG pillars at different molding temperatures under a pressing force of 1362 N.

The area ratios of the microdimples increase approximately logarithmically with the increase of molding temperature, as shown in Figure 16. By extrapolating the trend line in Figure 16, the temperature for preventing microdimple generating is approximately 379.8 °C. However, this temperature is too low and could lead to surface scratching. Therefore, it is inferred that the optimum temperature would be in the range of 380–382 °C [53].

**Figure 17:**
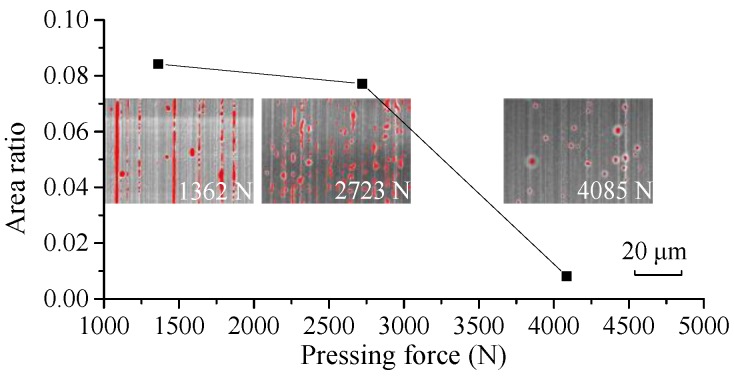
Area ratios of the microdimples of the formed ChG pillars under different pressing forces at 382 °C.

As shown in Figure 17, the area ratios of the microdimples decrease with the increase of the pressing force at a molding temperature of 382 °C. They decrease slowly from 1362 N to 2723 N and then continue to decrease rapidly from 2723 N to 4085 N. Therefore, if the contact pressure is larger than the saturated vapor pressure of the ChG, the gas will no longer be generated.

**Figure 18:**
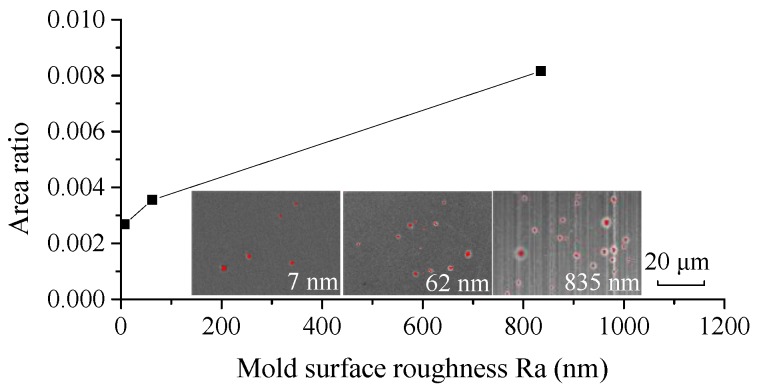
Area ratios of the microdimples of the formed ChG pillars with different mold surface roughness values under the pressure force of 4085 N at 382 °C.

Due to the surface microstructures of the mold and glass, the enclosed spaces result in a lower local pressure. The pressure of the enclosed spaces is lower than the saturated vapor pressure of ChG, which intensifies the evaporation of selenide gases, leading to incomplete reproduction and the formation of microdimples. Simultaneously, when the contact surfaces are smoother, the gas escapes more completely, which can result in a better surface quality [53]. Figure 18 shows the area ratios of the microdimples with different mold surface roughnesses. It can be concluded that smoother mold surfaces lead to fewer microdimples, which verifies the effect of the contact surface roughness on surface quality of molded ChG microstructures.

**Figure 19:**
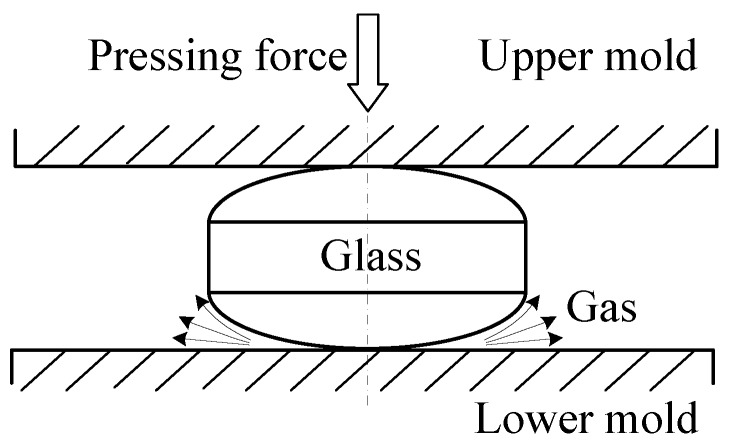
Schematic diagram of gas escape mode during the spherical glass molding process.

**Figure 20:**
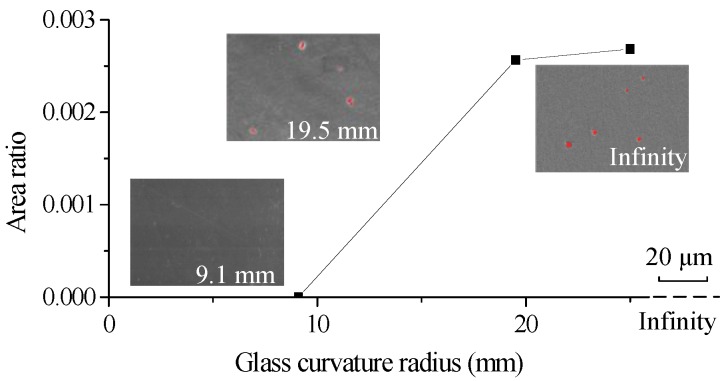
Area ratios of the microdimples of the formed ChG pillars with different glass surface curvature radii under 4085 N at a molding temperature of 382 °C.

When the ChG surface is curved with a smaller radius, the gas escape tends to be easier, as shown in Figure 19, and the area of contact surface is smaller when the pressing displacement stays the same, which means the pressure is larger under the same force with the same displacement. Hence, smaller radius ChG surface can reduce microdimples. It can be seen from Figure 20 that the area ratios of the microdimples of the formed pillars decrease, or are even completely eliminated, with the decrease of the glass curvature radius.

### 3.2. Forming Accuracy and Surface Quality Control

**Figure 21:**
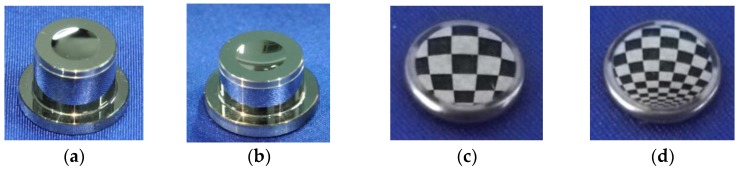
Photographs of aspherical molds and aspherical lens: (**a**) upper mold, (**b**) lower mold, (**c**) upper surface of aspherical lens, and (**d**) lower surface of aspherical lens.

The forming accuracy of aspherical lens impacts the optical properties of infrared optics, and the aspherical lens molding experiments are carried out to obtain high forming accuracy and surface quality. The precision aspherical molds and a molded ChG aspherical lens are shown in Figure 21. The formed lenses have an excellent surface finish of Ra 8 nm, and the surface profiles of formed lenses are consistent with the designed values.

**Figure 22:**
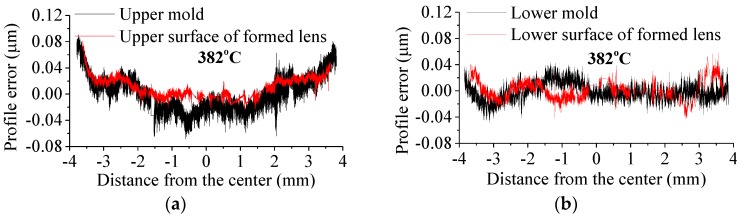

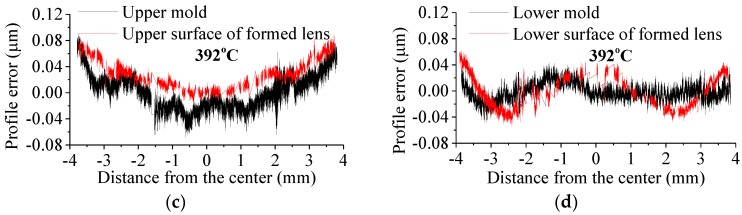
Evaluations of surface topography comparison of profile error: (**a**) between upper mold and lens formed at 382 °C, (**b**) between lower mold and lens formed at 382 °C, (**c**) between upper mold and lens formed at 392 °C, and (**d**) between lower mold and lens formed at 392 °C.

To demonstrate the conformity and replication fidelity of the formed lenses, the profile errors between molds and formed lenses are compared, as shown in Figure 22. Both the upper and lower finish surfaces of formed lenses are better than the molds, because the formed lens does not fill into the micro cavities caused by surface asperities of molds. However, higher molding temperature leads to better conformation between mold and glass surface, and larger roughness of the molded lens, indicating that the surface finish of the molded lens can be improved at lower molding temperature. Besides, the radius of curvature of the formed lens is less than the molds due to the shrinkage of glass in annealing and cooling stages. There is a shrinkage-inducing depression in the lower surface center of formed lens, where the cooling rate is larger than other places on this lens. Additionally, this shrinkage becomes greater with the increase of molding temperature.

Hence, the surface roughness of the formed aspherical lens can be improved by using a lower forming temperature. When replicating the specific micromorphology on the mold surface, the forming accuracy can be improved by increasing the mold temperature. Due to the shrinkage, there is also a difference between the profile of the formed aspherical lens and the mold. In the mold profile design, the shrinkage should be compensated.

## 4. Molding Process of ChG for Freeform Optics

### 4.1. ChG Molding for Microlens Array

Although silicon carbide (SiC) and tungsten carbide (WC) are preferable mold materials owing to their high hardness and toughness under molding conditions, it is quite difficult to generate microlens array and microstructures on these materials [57,58]. In order to extend the PGM to creating microlens array and microstructures on the ChG surface, the mold should be machined with high accuracy and efficiency. Electroless nickel phosphorus (Ni-P) has emerged as an outstanding hard coating material because of its great hardness, outstanding corrosion resistance, and antiwear property [59,60,61]. Therefore, Ni-P plating has been used as mold to fabricate microlens array and microstructures for PGM [62,63].

**Figure 23:**
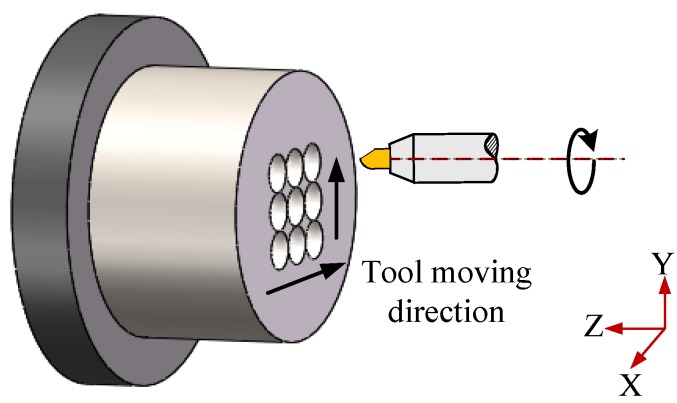
Single point diamond cutting process of ChG microlens arrays.

In the microlens arrays mold preparation, the Ni-P layer is coated on the substrate through electro-less plating method, and the upper and lower molds are processed by diamond cutting, using a round diamond tool. Then, microlens arrays are generated on the lower mold by using a high-speed diamond-ball nose-end milling tool with an included fillet radius of 500 μm and a shank diameter of 6 mm (in Figure 23).

**Figure 24:**
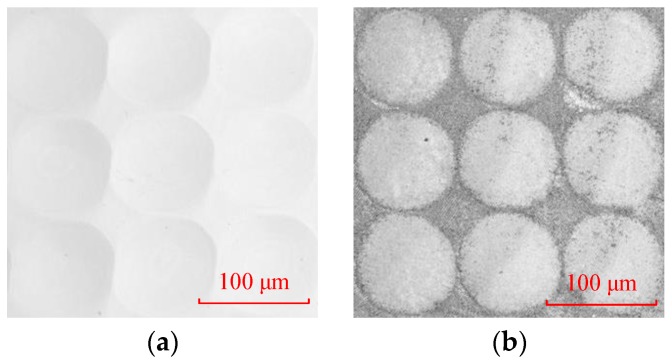
Microscope images of microlens arrays: (**a**) microlens arrays on the mold and (**b**) microlens arrays on the ChG.

**Figure 25:**
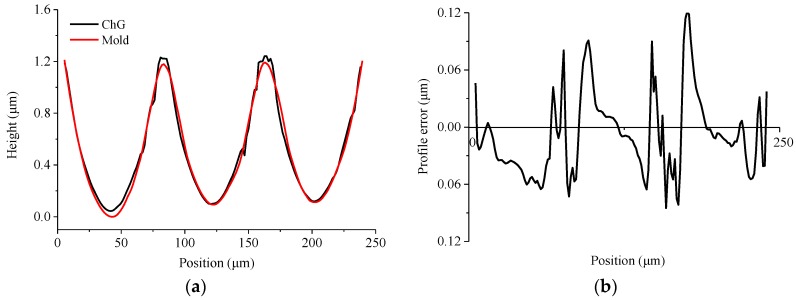
The profile curves of microlens arrays: (**a**) microlens arrays on the mold and the ChG, and (**b**) profile error of microlens arrays.

Figure 24a is the microscopic image of microlens arrays on the Ni-P mold. The diameter of the microlens is 80 μm, and the height of the microlens is 1 μm. Figure 24b is the microscopic image of microlens arrays on the ChG. To demonstrate the conformity and replication fidelity of microlens arrays, the microlens arrays profiles of mold and ChG are compared, and the profile error is analyzed by the least square method (shown in Figure 25). The peak-to-valley height error is 0.121 μm, and the average error of the height is 0.034 μm, which meet design requirements.

### 4.2. ChG Molding for Microstructures

**Figure 26:**
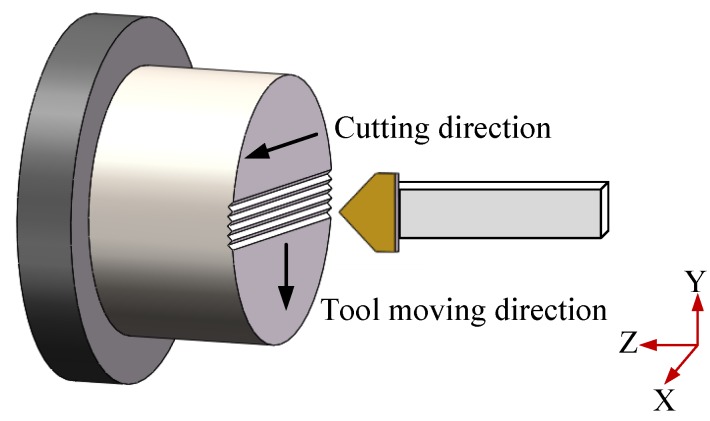
Single point diamond turning process of ChG microstructures.

Before the microstructures ChG molding, mirror mold surfaces are generated on the Ni-P plating layer by diamond cutting using a round diamond tool. Moreover, microstructures are generated using a triangle diamond tool with an included angle of 90° by feeding the diamond tool on the lower mold with three-axle linkage. Therefore, microstructures with more complicated geometric are formed with high accuracy, as shown in Figure 26.

**Figure 27:**
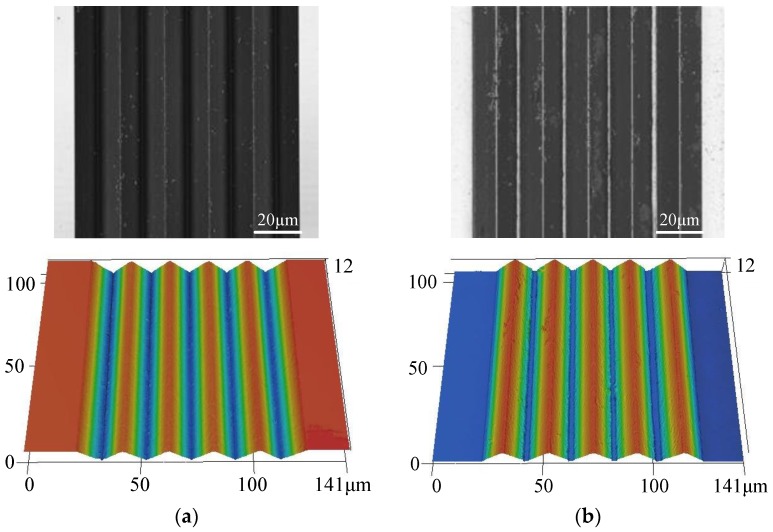
Microscope images of microstructures on (**a**) mold surface and (**b**) ChG surface.

**Figure 28:**
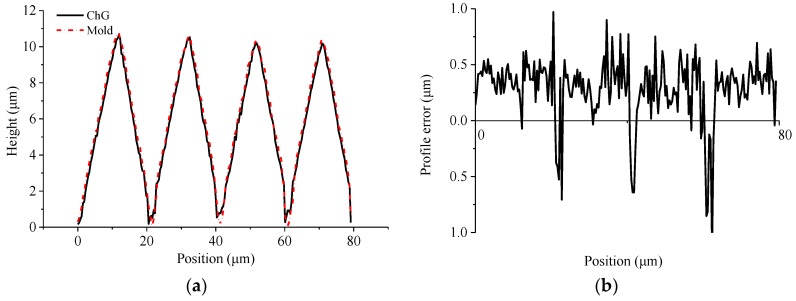
The profile curves of microstructures: (**a**) microstructures on the mold and the ChG, and (**b**) profile error of microstructures.

Microscopic observations of the ChG surface and the mold surface are performed using a confocal laser scanning microscope, and the microscopic observations of the microstructures are shown in Figure 27. The contour curves of the microstructures on the ChG surface and mold surface are extracted to analyze the forming quality, as shown in Figure 28. The average error of the height is 0.36 μm, and the peak-to-valley height error is 0.97 μm. It can be seen from the contour curve that the top of the microgroove is sharp, although the forming quality of bottom is slightly worse compared with that of the top. Therefore, PGM can achieve high replication accuracy of the ChG microstructures. Moreover, the minimum feature size of ChG microstructures is limited by the size of microstructures on molds, and the ChG molding can produce optics with feature sizes from millimetres to micrometres to nanometres.

**Figure 29:**
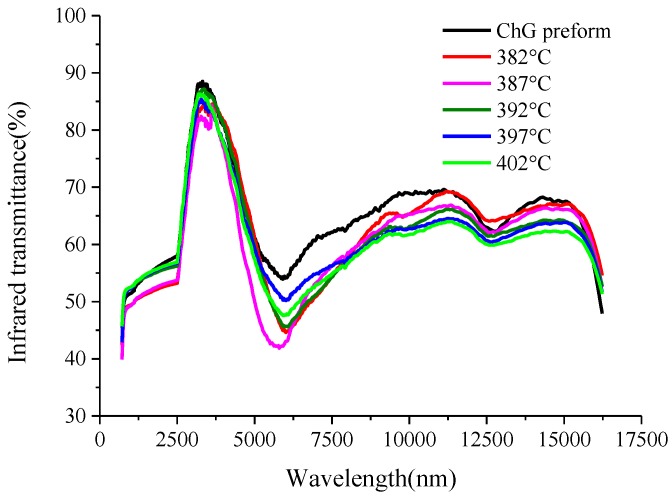
Infrared transmittance of the formed ChG at different molding temperatures.

Raman spectroscopy detections of ChG preform and formed ChG made into 2 mm sheets are carried out to study the influence of molding process on infrared transmission characteristics of ChG, as shown in Figure 29. Infrared transmittances of the ChG formed at various molding temperatures are almost identical to ChG preform, and the infrared transmission wavelength band of them are 0.8–16 μm. Though the transmittance of ChG is low at the wavelength of 6 um due to the absorption band of oxide impurities in ChG, it covers three atmospheric windows of 1–3 μm, 3–5 μm, and 8–12 μm, which are generally used in infrared systems. Therefore, ChG infrared optics fabricated by PGM meet the requirements of infrared optical systems.

## 5. Innovations of ChG Molding

### 5.1. Localized Rapid Heating Process for ChG Molding

PGM can achieve mass production of ChG optics with a good forming accuracy. However, conventional PGM is a bulk heating process that usually requires a long thermal cycle. The molding assembly and ChG are heated and cooled together, which often cause large thermal expansion in both the molds and ChG infrared optics. A localized rapid heating process is developed to effectively heat only bottom surface of the ChG. Graphene film coated on the mold surface only elevates the temperature of area where microlens array and microstructures need to be replicated, which can reduce the undesired thermal expansion of the mold and ChGs [64,65].

**Figure 30:**
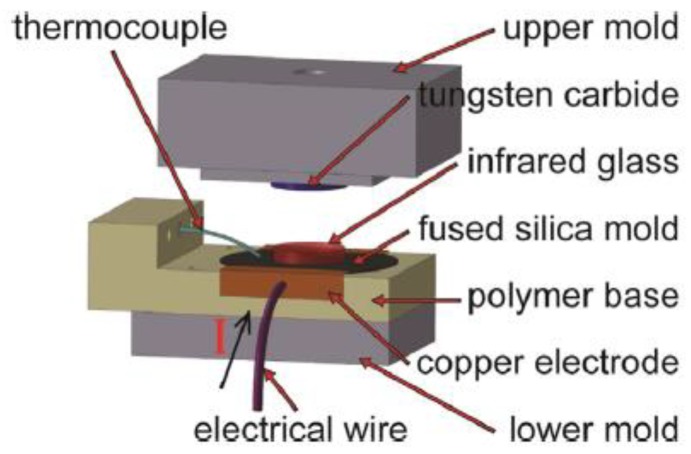
The localized rapid heating molding assembly [65].

**Figure 31:**
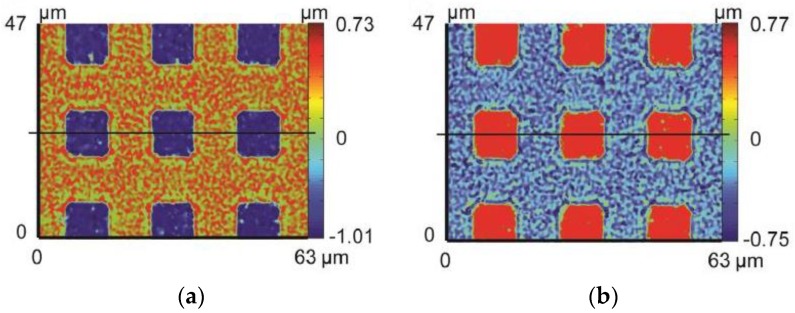
Microscope images of microstructures on (**a**) mold surface and (**b**) molded ChG surface after localized rapid heating process [65].

Localized rapid heating glass molding assembly is shown in Figure 30. A specially designed polymer base plate is fixed on the lower mold, placing under the graphene-coated, fused silica wafer and a thermocouple mounted on the fused silica wafer. Two copper electrodes are sandwiched on the front and back edges of the polymer base plate. The lower mold is moved up by a linear drive until the top surface of the glass touched the bottom of the tungsten carbide. A direct current (60 V/0.84 A) is applied on the copper electrodes during heating stage. The surface of the fused silica wafer is heated up rapidly. Nitrogen flow is introduced into the chamber to accelerate the cooling during annealing and cooling stages. Afterwards, the load is released and the formed ChG is removed from the chamber. The molded surface of the ChG using localized rapid heating and the mold surface are shown in Figure 31. It can be confirmed the ChG fills precisely into microstructures on mold by localized rapid heating process.

### 5.2. Contactless Molding of ChG Microlens Array

Most of ChGs have a high content of unstable volatile elements, and these elements tend to diffuse out from performs at high temperature and deposit on the surface of the molds, which induce severe interfacial adhesion problems and make it more difficult for molding process. Therefore, gas-assisted contactless molding process is developed to form microlens array avoiding interfacial adhesion problems [66].

**Figure 32:**
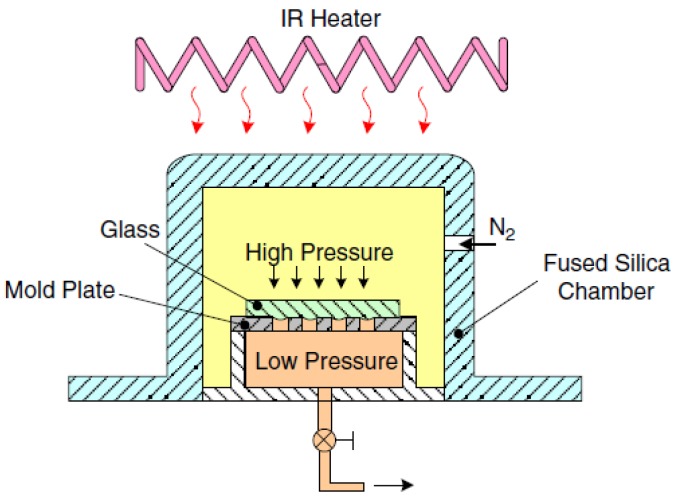
Schematic diagram of gas pressure-assisted glass molding machines [66].

The gas-assisted contactless molding processes machine is shown in Figure 32. The IR heater is placed on the top of the fused silica chamber with programming control for the molding temperature. The position of the temperature sensor is located outside the chamber but directly contacts the quartz tube near the sample. The stainless steel plate with arrayed through holes is used as a mold. The heating temperature is gradually increased to approach the softening point to allow the glass plate to be driven downward by the N_2_ gas pressure, so that the ChG flows into the arrayed through holes and forms the microlens array.

**Figure 33:**
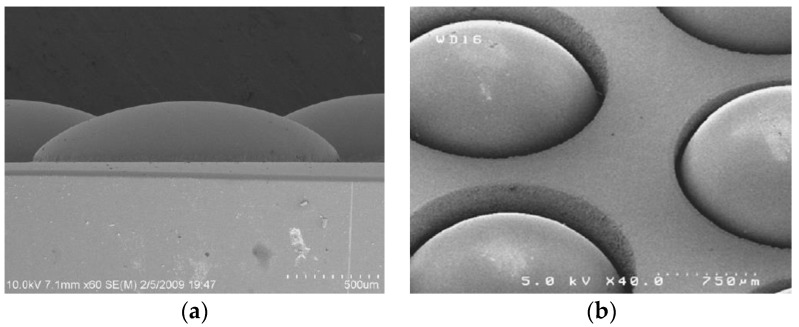
SEM images of ChG microlens array: (**a**) the cross-sectional image of the molded ChG microlens array and (**b**) the appearance of molded ChG microlens array [66].

The SEM images of ChG microlens array are shown in Figure 33. Hence, the contactless gas-assisted molding system can avoid contact-induced glass sticking and gas bubble problems. Besides, the surface profile of the molded lenses can be precisely controlled by changing the applied gas pressure, molding temperature, and time duration.

## 6. Outlook

In this article, we have overviewed recent progresses in infrared optics fabrication by PGM. To date, some part of them, like elastic-viscoplasticity constitutive model and ChG molding surface defect reduction, are relatively mature. However, several hot topics, such as atomic diffusion, interfacial adhesion, and anti-reflection film technology, still need further study [67,68,69].

(1) Atomic diffusion

Atomic diffusion will come up between the surfaces of ChG and mold in molding process. The motion of atoms is violent, and the diffusion phenomenon is serious at high temperature, which will lead to the change of optical property of ChG and thermal mechanical property of mold material. The diffusion degree of atoms is related to the surface energy and surface quality of the mold materials [70]. Therefore, the influence of the mold materials on atomic diffusion is necessarily studied to choose the suitable mold material.

(2) Interfacial adhesion

Interfacial adhesion has been a major challenge in PGM. It results in the surface quality deterioration of the mold and shortens the service life of the mold, which escalates the overall cost and decreases the repeatability. Protective coatings, such as diamond-like carbon (DLC), platinum-iridium (Pt-Ir), rhenium-iridum (Re-Ir), and so on have been employed to prevent interfacial adhesion [71,72]. These coatings have low friction coefficient and antisticking property. However, the alternating stress during repeated thermal cycles in PGM will cause coatings to peel off. It is urgent to study the mechanism of interfacial adhesion and develop suitable techniques to prevent interfacial adhesion.

(3) Anti-reflection film technology

The elements of sulfur S, selenium Se, and tellurium Te in ChG are easily influenced by environment. Meanwhile, the ChG has high refractive index and large reflection loss. The refractive index of ChG will reduce after PGM [73]. In order to improve the infrared optical performance of the ChG, anti-reflective film technology needs to be further explored to effectively reduce the infrared reflection loss of ChG and improve the infrared optical transmittance. Hence, an anti-reflective coating on the ChG infrared optics should be applied after molding, and the maximum utility of ChG infrared optics can be achieved in the infrared systems.
